# A Smooth-Motion Walking-Type Piezoelectric Actuator over a Large Travel Range with High Torque

**DOI:** 10.3390/biomimetics11070495

**Published:** 2026-07-14

**Authors:** Jianfei Cheng, Weishan Chen, Jianhua Sun, Jinghan Guan, Mingxin Xun, Shijing Zhang, Jie Deng, Yingxiang Liu

**Affiliations:** State Key Laboratory of Robotics and System, Harbin Institute of Technology, Harbin 150001, China; jf_cheng@hit.edu.cn (J.C.); cws@hit.edu.cn (W.C.); 22b908004@stu.hit.edu.cn (J.S.); 21b908004@stu.hit.edu.cn (J.G.); 21b908006@stu.hit.edu.cn (M.X.); zhangshijing@hit.edu.cn (S.Z.)

**Keywords:** piezoelectric actuator, smooth motion, high torque, bipedal walking gait

## Abstract

Piezoelectric actuators are widely used in precision manufacturing, high-end equipment and aerospace fields due to their high precision, fast response and flexible structure. However, continuous smooth displacement output over a large travel range with high-torque requirement is still a problem that needs to be solved, which greatly limits their application scope. Therefore, this work proposes a smooth-motion walking-type piezoelectric actuator over a large travel range with high torque inspirited by the bipedal walking mechanism. The driving legs with high stiffness and large displacement of the actuator are divided into two groups; by imitating the bipedal walking gait of humans, the driving trajectories of the two sets of driving legs are alternately integrated to adjust the balance between the driving torque and the resistance torque, ensuring that the resultant force torque acting on the rotor is zero, thereby achieving smooth motion in a large travel range with high torque. The multi-objective optimization algorithm of the neighbourhood cultivation genetic algorithm (NCGA) is adopted to optimize the structure design of the driving legs by considering the stiffness and displacement, in order to achieve the balance of high stiffness and large displacement at the foot end of the driving leg. The linear fitting coefficients of the output displacements of the actuator all reach above 0.999, the resolution is 0.4 µrad, and the maximum output torque is better than 1.31 N·m. These performances will greatly expand the application fields of piezoelectric actuators, especially in deep space optical tracking scenarios.

## 1. Introduction

Piezoelectric actuators are widely used in aerospace [1,2,3], biomedical field [4,5,6], and robot joints [7,8,9,10], among other fields [11,12,13,14], due to their advantages such as high precision, fast response, flexible structure and no electromagnetic interference characteristic [15,16,17,18,19,20]. They can be classified into direct driving type [21,22,23], inertial type, inchworm type [24,25,26] and ultrasonic type [27,28,29] according to the actuation principle. The direct driving type has the characteristics of simple structure and high resolution, but the output travel is limited [30,31,32]. Although the inertial type has a simple excitation signal, its output displacement exhibits rollback, which limits large output force and smooth motion [33,34,35,36]. The inchworm actuating type has high positioning accuracy and a large travel range. However, its structure is complex and its stair-type output displacement leads to its unsmooth motion [37,38,39,40,41]. The ultrasonic type has the characteristics of high speed and large output force, but it suffers from severe wear and tear, leading to a speed fluctuation problem under long-term operation [42,43,44,45,46]. It can be concluded that it is a challenge to achieve smooth displacement output over large travel ranges for the piezoelectric actuators, especially under the high-torque and low-speed conditions.

There are two approaches for the piezoelectric actuators to achieve smooth motion over large travel ranges: the design of a proper configuration and an exciting scheme. Researchers have designed a series of configurations to realize the smooth motion of the actuator. For example, Liu et al. proposed a stick-slip piezoelectric actuator with a dual-stator cooperative actuation mode, which suppressed the rollback motion of the rotor [47]. Dong et al. proposed a stick-slip linear piezoelectric actuator. During the reverse driving process, the reverse motion is actively suppressed by clamping the slider through the active locking mechanism. The maximum output force is 1.6 N at 100 V [48]. Tian et al. utilized passive damping feet to suppress the backward movement of the mover, achieving a load capacity of 9.33 kg under the conditions of 741 g load and driving frequency at 200 Hz, and the resolution of the actuator at 4.61 nm [49]. Ding et al. achieved smooth motion of the actuator by alternately stepping two driving feet through a piezoelectric stack [50,51]. The actuator demonstrated excellent smoothness under both no-load and horizontal load conditions, with resolutions at 0.80 µm [50]. Ji et al. integrated an L-shaped displacement amplification mechanism and a parallel quadrilateral compliance mechanism on the actuator. By dynamically adjusting the preload between the fixed and moving elements, the single-step displacement is effectively increased, achieving smooth motion output. The maximum load capacity is 2.1 N [52].

In addition, a large number of researchers have made many improvements in the exciting scheme to achieve smooth motion of the actuator. For example, Xu et al. proposed an active and passive cooperative driving method. The actuator exhibits highly linear and smooth displacement within the range of 50 V to 100 V. When the horizontal load remains below 30 g, the actuator achieves exceptionally smooth and linear motion [53]. Yu et al. proposed a collaborative incentive method to eliminate rollback and improve the smoothness of motion. The linearities of the motion of the actuator in the X and Y directions were 99.995% and 99.994%, respectively. It can achieve an infinite motion range and the motion resolution is higher than 3.96 nm, while its stable load capacity is greater than 15.6 times of its own weight [54]. Qiu et al. proposed a method of cooperative compensation using two signals with specific initial time intervals to reduce rollback. This excitation method can effectively increase the step size of the actuator, especially under low-frequency driving. The step size of the actuator using this excitation method is more than 29 times that of the traditional single-driving method at 10 Hz. When the signal frequency is 250 Hz, the maximum step size of the actuator is 4.448 µm using the excitation method, which is over 40% that of the traditional single-driving method [55]. Based on the bent-bend hybrid inertial actuator, Cheng et al. proposed a step-consistent active control method. This method utilizes embedded strain gauge as a force sensor to maintain constant pressure between the mover and the actuator, achieving the maximum standard deviation of the step within 2 mm travel range at 0.41 µm when the driving conditions are 350 V_p-p_ and 1 Hz, while under the same conditions of non-active control, the maximum standard deviation of the step is 1.14 µm [56]. Ning et al. proposed a bimodal excitation method, which utilizes piezoelectric stack and two piezoelectric plates as the main and auxiliary excitation sources of the actuator respectively. High-frequency sine waves are applied to the piezoelectric ceramic plates to generate ultrasonic vibrations, and the backward movement of the driving foot is suppressed through ultrasonic friction reduction. Through this method, the rollback rates in the forward and reverse directions were reduced by 10% and 5% respectively, the speeds were increased by 17% and 9% respectively, and the loads were increased by 13% and 11% respectively [57].

Although the above-mentioned piezoelectric actuator has achieved smooth motion over large travel ranges, it is still a challenge to achieve smooth motion under high torque over large travel ranges. This is mainly because they achieved smooth motion through improvements in configuration and excitation methods without simultaneously enhancing the contact stiffness between the stator and the rotor. The high torque output over large travel ranges cannot be obtained since there are strict requirements for contact force between the stator and the rotor, and the speed fluctuation problem occurs in the long-term due to the insufficient contact stiffness and the processing and assembly accuracy of the contact surface. Therefore, this work proposes a multi-legged walking-type piezoelectric actuator with high torque and smooth motion. The driving legs with high stiffness and large displacement of the actuator are divided into two groups. By imitating the walking gait of humans, the actuation trajectories of the two groups of driving legs are alternately integrated to adjust the balance between the driving torque and the resistance torque, ensuring that the resultant torque acting on the rotor is zero, thereby achieving smooth motion. The interaction effect and main effect between the design variables and the design objectives were considered simultaneously during the multi-objective optimization process, enabling the driving legs to take into account the characteristics of large displacement and high stiffness, thus enhancing the contact stiffness between the stator and the rotor. The smooth motion over a large travel range can be achieved under the high-torque and low-speed conditions. The detailed structure of this work is as follows: Section 2 introduces the mechanism and actuation principle of the actuator. Section 3 introduces the analysis of design variables and the optimal design of actuators. Section 4 introduces experimental results of the displacement characteristics of the driving foot and the resolution, and smoothness characteristics and torque characteristics of the actuator to verify the effectiveness of the design method. Through these various works, the proposed stepping piezoelectric actuator is designed, developed, and verified to have the ability to achieve smooth motion over a large travel range and high torque output.

## 2. Structure and Mechanism

In this section, the overall configuration of the piezoelectric actuator is illustrated first, and then the principle of smooth motion is illustrated.

### 2.1. Configuration and Assembly of Actuators

To ensure smooth motion of the piezoelectric actuator, the resultant external torque applied on the rotor at any moment should be zero. Therefore, it is necessary to guarantee that there is static friction between the stator and the rotor, as static friction driving can ensure that the driving torque of the stator acting on the rotor at any time is balanced with its own resistance torque. This requires that the actuator has more than two sets of driving feet for alternating driving, and the driving feet can effectively contact and separate from the rotor, with the driving speeds of different sets of driving feet remaining consistent. Considering the structural stability provided by triangular configurations, each actuation unit in the proposed design incorporates three driving legs. Consequently, the six driving legs are partitioned into two actuation units.

**Figure 1:**
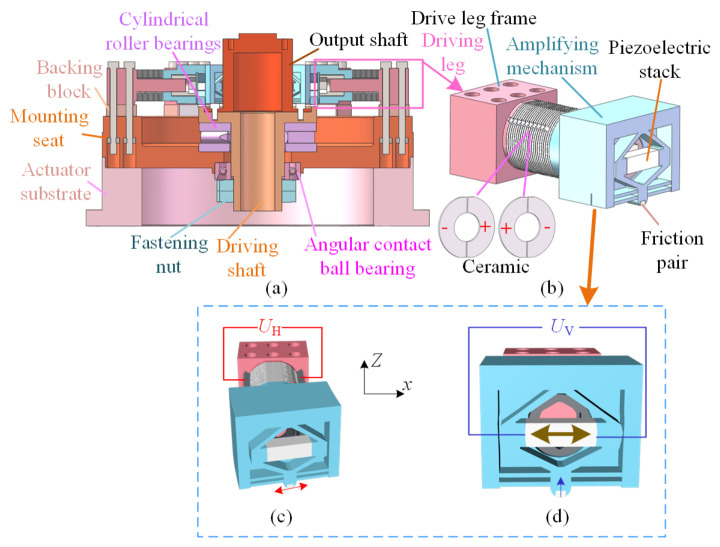
Structure and configuration of the actuator. (**a**) A sectional view of the overall configuration of the actuator. (**b**) The configuration of the driving leg. (**c**) Schematic diagram showing the deformation of the foot end along the driving direction when the voltage is applied to the ceramic. (**d**) Schematic diagram showing the deformation of the foot end along the driving direction when the voltage is applied to the stack structure.

The piezoelectric actuator comprises an actuator substrate, a mounting seat, six driving legs with associated installation modules, and a shaft system. Each driving leg installation module is assembled with six screws and backing blocks. As detailed in Figure 1 and Video S1, the driving leg consists of: 22 two-zone d_33_-mode piezoelectric ceramics (the ceramic size is Ø30 × Ø14 × 1 mm, with a thickness of 0.1 mm of brass sheets arranged between the ceramics), a driving leg frame, an amplification mechanism, a piezoelectric stack (the stack size is 7 × 7 × 20 mm, from China Core Tomorrow Technology Co., Ltd., Harbin, China), and a friction pair. During the driving process, the leg foot ends of the driving leg undergo bending movements along the *x*-axis (as shown by the red line in Figure 1c) and clamping movements along the *Z*-axis (as shown by the blue line in Figure 1d). The shaft system incorporates a driving shaft, two fastening nuts, one angular contact ball bearing (7010AC, from Luoyang Zhongyue Precision Co., Ltd., Luoyang, China), one cylindrical roller bearing (87410-TV, from Schaeffler Co., Ltd., Herzogenaurach, Germany), and an output shaft; the use of angular contact ball bearings in the shaft system ensures that the shaft system has high stiffness.

### 2.2. Operating Principle

**Figure 2:**
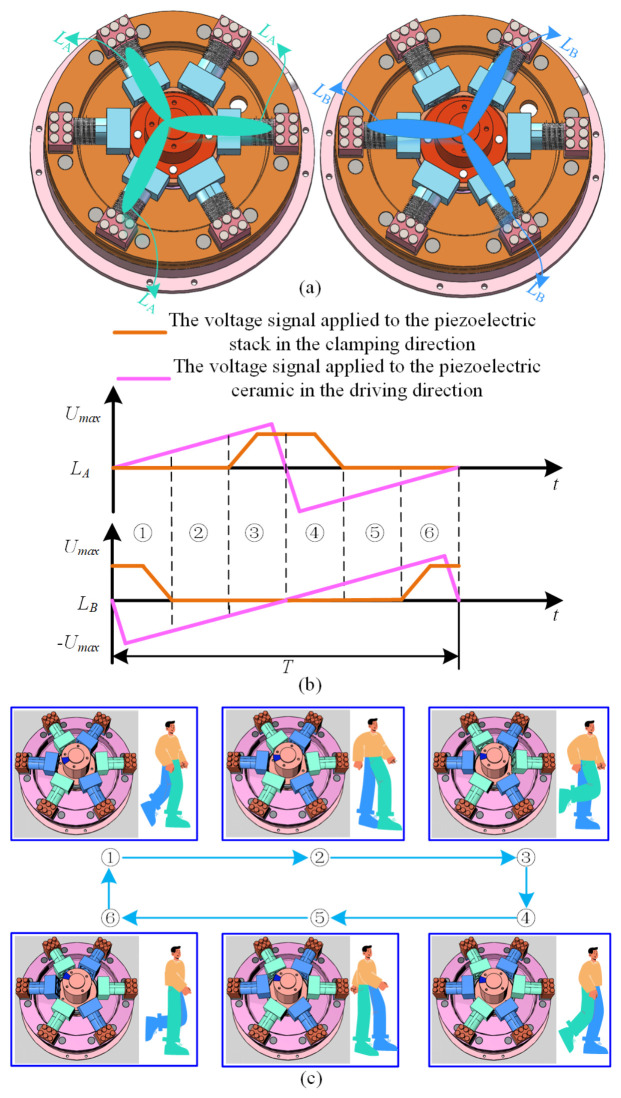
Working principle of piezoelectric actuators. (**a**) Classification of actuation feet. (**b**) Excitation signal. (**c**) Phase sequence of rotor actuation.

The driving legs are divided into two groups, with each group consisting of three legs. The large torque output is achieved through two methods: the parallel driving of multiple driving legs and the enhancement of the contact stiffness between the driving legs and the rotor, as illustrated in Figure 2a. Each group of driving units is equivalent to one leg of a human, as shown in Figure 2b. According to the walking gait of a human, an actuation cycle is divided into six steps. The excitation signals applied to the actuator are presented in Figure 2b, and the actuation sequence of the actuator is depicted in Figure 2c. The motion sequence enabling the smooth motion of the piezoelectric actuator is presented in Video S1 and detailed below:

Step 1: At this stage, the left leg of a man gradually touches the ground from the lifted position, while the right leg remains in contact with the ground. Corresponding to the actuator, the clamping-direction voltage of the group A driving unit is 0 V, and the driving leg in contact with the rotor. The driving-direction voltage of the group A driving unit gradually increases from 0 V to the maximum forward voltage. The clamping-direction voltage of the group B driving unit first maintains the maximum value and then gradually decreases to 0 V. The driving legs with the rotor gradually change from the separated state to the contact state. The driving-direction voltage of the group B driving unit gradually increases from 0 V to the negative maximum value and then gradually decreases. At this stage, group A driving units drive the rotor, while group B driving units gradually come into contact with the rotor.

Step 2: At this stage, the left leg of a man is in contact with the ground, and the right leg is in contact with the ground. Corresponding to the actuator, the clamping-direction voltage of the group A driving unit is 0 V, and the driving leg is in contact with the rotor. The driving-direction voltage of the group A driving unit continues to increase towards the maximum value in the forward direction. The clamping-direction voltage of the group B driving unit is 0 V, and the driving leg is in contact with the rotor. The driving-direction voltage of the B drive unit continues to gradually decrease from the negative to the maximum value. At this stage, both group A and group B driving units drive the rotor.

Step 3: At this stage, the left leg of a man is in contact with the ground while the right leg is separated from it. Corresponding to the actuator, the voltage in the clamping direction of the group A driving unit gradually increases from 0 V to the maximum voltage. The driving leg with the rotor changes from the contact state to the separated state. The voltage in the driving direction of the group A driving unit continues to increase towards the maximum voltage in the forward direction. After increasing to the maximum voltage, it gradually decreases to 0 V. The clamping-direction voltage of the group B driving unit is 0 V, and the driving leg is in contact with the rotor. The driving-direction voltage of the group B driving unit continues to gradually increase from the negative maximum value to 0 V. At this stage, group A driving units gradually separate from the rotor, while group B driving units drive the rotor.

Step 4: At this stage, the left leg of a man is in contact with the ground, and the right leg gradually comes into contact with the ground. Corresponding to the actuator, the clamping-direction voltage of the group A driving unit first maintains the maximum voltage and then gradually decreases to 0 V. The driving leg with the rotor gradually changes from the separated state to the contact state. The driving-direction voltage of the group A driving unit first increases from 0 V to the negative maximum value and then gradually decreases. The clamping-direction voltage of the group B driving unit is 0 V, and the driving leg is in contact with the rotor. The driving-direction voltage of the group B driving unit gradually increases from 0 V to the maximum forward value. At this stage, group A actuator units gradually come into contact with the rotor, while group B driving units drive the rotor.

Step 5: At this stage, the left leg of a man is in contact with the ground, and the right leg is in contact with the ground. Corresponding to the actuator, the voltage in the clamping direction of the group A driving unit is 0 V, and the driving leg is in contact with the rotor. The voltage in the driving direction of the group A driving unit continues to gradually decrease towards the negative maximum value. The clamping-direction voltage of the group B driving unit is 0 V, and the driving leg is in contact with the rotor. The driving-direction voltage of the group B driving unit continues to increase towards the maximum forward voltage. At this stage, both group A and group B driving units drive the rotor.

Step 6: At this stage, the left leg of a man is separated from the ground while the right leg is in contact with it. Corresponding to the actuator, the clamping-direction voltage of the group A driving unit is 0 V, and the driving leg is in contact with the rotor. The driving-direction voltage of the group A driving unit continues to gradually decrease from the negative maximum value to 0 V. The clamping-direction voltage of the group B driving unit gradually increases from 0 V to the maximum value. The driving leg and the rotor gradually change from the contact state to the separated state. The driving-direction voltage of the group B driving unit first increases to the forward maximum value and then gradually decreases to 0 V. At this stage, group A driving units drive the rotor, while group B driving units gradually separate from the rotor.

Repeating steps 1 to 6 can achieve an infinite range of motion. Changing the direction of the horizontal driving signal can achieve reverse movement.

## 3. Mechanical Design and Optimization of Actuation Legs

**Figure 3:**
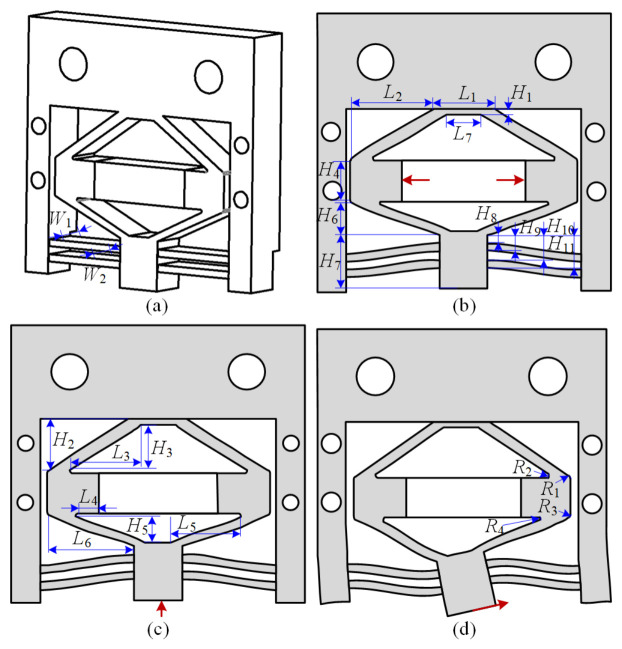
Schematic diagrams of the flexible compliant bistable mechanism under different bending modes. (**a**) Schematic diagram without external influence. (**b**) Schematic diagram when voltage is only applied to the stack. (**c**) Schematic diagram of applying external force only in the direction of clamping at the foot end of the driving leg. (**d**) Schematic diagram of applying external force only in the driving direction at the foot end of the driving leg.

According to the actuator action sequence planned in Section 2, it can be known that the driving leg of the actuator needs to have the decoupling motion capability in both the driving direction and the clamping direction simultaneously for achieving smooth motion. In addition, the contact stiffness between the stator and the rotor is the key to achieving high torque. This requires both the stator and the rotor to have high stiffness characteristics, and the rotor achieves high stiffness by using cylindrical roller bearings. Therefore, it is necessary to optimize the driving direction stiffness and clamping direction stiffness of the driving legs. The structural dimension of the driving direction of the driving leg has been optimized. Corresponding to the smooth motion mentioned earlier, the mechanism needs to have high stiffness. The stiffness of the driving mechanism in [54] meets the requirements, but the clamping mechanism needs to be designed with high stiffness and large displacement. The purpose of large displacement in the clamping direction is to be able to disengage the driving leg from the rotor. In order to improve the efficiency of optimization calculations, the flexible compliant bistable mechanism is simplified, as shown in Figure 3. From Figure 3, it can be seen that the flexible compliant bistable mechanism has 24 size parameters. If all size parameters are used as variables for optimization, on the one hand, the computational efficiency is low; on the other hand, too many parameters can easily lead to structural dimension interference and make it difficult to obtain optimization results. Therefore, it is necessary to conduct sensitivity and interaction effect analysis on the parameters, reduce the dimension of the parameters to determine the key design variables, and then carry out optimization design based on the determined key design variables.

### 3.1. Critical Parameter Sensitivity Analysis

When optimizing the dimension of the rhombic amplification mechanism, the displacement in the clamping direction, the stiffness in the clamping direction and the stiffness in the driving direction need to be taken into consideration. The displacement in the clamping direction determines whether the driving leg can effectively contact and separate from the rotor, the stiffness in the clamping direction determines the output torque of the actuator, and the stiffness in the driving direction determines the displacement coupling in the horizontal direction when the driving leg contacts the rotor. The displacement in the clamping direction is the displacement of the foot end of the driving leg when the voltage of the piezoelectric stack is driven at 150 V. In order to ensure the convenience of sample design and analysis, the displacement in the clamping direction generated at the foot end of the driving leg under preload of 90 N is taken as the equivalent stiffness value (when the preload force is 60 N, the output torque of the actuator is 1 N·m. With the safety factor of 1.5, the corresponding applied preload force is 90 N). Since the friction coefficient between the foot end of the driving leg and the rotor is 0.13, it is necessary to apply an external force of 11.7 N in the driving direction at the foot end of the driving leg, solve for the horizontal displacement, and then calculate the stiffness value in the driving direction.

**Figure 4:**
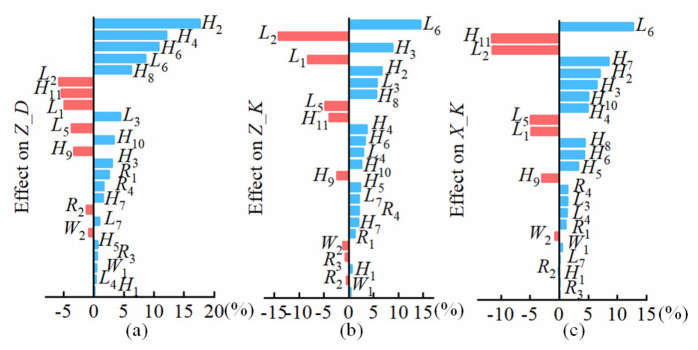
Analysis of the interaction effects of each design variable: (**a**) Analysis of the interaction effect of clamping direction displacement (*Z_D*). (**b**) Analysis of the interaction effect of the equivalent value of the clamping direction stiffness (*Z_K*). (**c**) Analysis of the interaction effect of the equivalent value of the driving direction stiffness (*X_K*).

**Figure 5:**
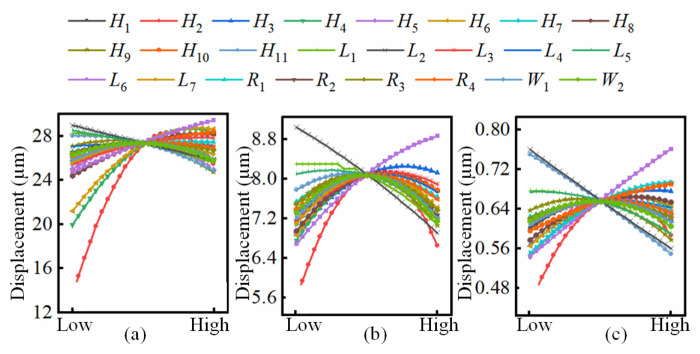
Analyze the main effects of each design variable: (**a**) Analysis of the main effect of clamping direction displacement (*Z*_*D*). (**b**) Analysis of the main effect of the equivalent value of the clamping direction stiffness (*Z*_*K*). (**c**) Analysis of the main effect of the equivalent value of the driving direction stiffness (*X*_*K*).

The Z_K represents the displacement of the foot end clamping direction of the driving leg under the action of an external force of 90 N, the X_K represents the displacement in the driving direction at the foot end of the driving leg under the action of an external force of 11.7 N, and the Z_D represents the displacement in the clamping direction at the foot end of the driving leg when the voltage on the stack is 150 V. For the 24 design variables, as shown in Table 1, −8% to 8% of their initial values were respectively selected as the value range of the design variables, and 600 iterative calculations were carried out using the optimized Latin square sample design method. Through post-processing, the interaction and main effect relationships between 24 design variables and the foot end displacement (Z_D) in the clamping direction of the driving leg, the equivalent value of the clamping direction stiffness (Z_K) and the equivalent value of the driving direction stiffness (X_K) can be obtained, as shown in Figure 4 and Figure 5 respectively.

As can be seen from Figure 4, the design variables have no interaction effect with the displacement in the clamping direction, the equivalent value of the stiffness in the clamping direction, and the equivalent value of the stiffness in the driving direction. Therefore, when selecting the key variables, the interaction effect among the design variables can be ignored. Based on the main effect data in Figure 5, the principle for selecting key variables is as follows:

(1) The design variable can increase *Z*_*D* while reducing *Z*_*K* and *X*_*K* (when *Z*_*K* and *X*_*K* decrease, the stiffness in the corresponding direction increases), or has little influence on *Z*_*K* and *X*_*K*.

(2) The design variable can reduce *Z*_*K* (when *Z*_*K* is reduced, the clamping direction stiffness increases) while increasing *Z*_*D* and reducing *X*_*K*, or has little influence on *Z*_*D* and *X*_*K*.

(3) The design variable can reduce *X*_*K* (the stiffness of the driving direction increases when *Z*_*K* decreases), and at the same time increase *Z*_*D* and decrease *Z*_*K*, or has little effect on *Z*_*D* and *Z*_*K*.

Based on the above three principles, the nine design variables *L*_1_, *L*_4_, *L*_5_, *H*_2_, *H*_6_, *H*_8_, *H*_9_, *H*_10_, and *H*_11_ are ultimately determined as the key design variables.

### 3.2. Structural Optimization

When designing the flexible mechanism of the driving leg, those factors such as processing and assembly need to be taken into account. For instance, the installation error between the cylindrical roller bearing and the mounting seat (6 µm), the surface runout of the cylindrical roller bearing (3 µm), and the runout of the contact surface between the foot end of the driving leg and the rotor (5 µm) results in a total tolerance of 14 µm caused by these three factor. If the safety factor of 1.5 is selected, the maximum runout of the working surface is 21 µm. Considering that when the foot end of the driving leg is in the rated preload state, applying a 150 V voltage on the piezoelectric stack with the displacement of the foot end in the clamping direction being greater than 21 µm can enable the driving leg to achieve contact and separation around the entire rotor when the displacement of the foot end in the clamping direction is taken as a constraint condition.

In addition, the greater the stiffness in the clamping direction and the driving direction, the more conducive to achieving smooth motion. Therefore, the maximization of the stiffness in the clamping direction and the driving direction is taken as the optimization objective, and the value range of the nine key design variables is −8% to 8% of the initial value. Based on the above optimized objectives and design variable constraints, the optimized model is established and expressed in Equation (1).

(1) $$\begin{array}{c} \max\left\{K_{Z}\left.,K_{X}\right\}\right.\\ S.T.\left\{\begin{array}{l} K_{Z}=90/Z\_K\\ K_{X}=11.7/X\_K\\ Z\_D\geq 0.000021+Z\_K\\ Z^{T}=\left[L_{1},L_{4},L_{5},H_{2},H_{6},H_{8},H_{9},H_{10},H_{11}\right] \end{array}\right. \end{array}$$

where *Kz* represents the stiffness in the foot end clamping direction of the driving leg, and *Kx* represents the stiffness in the driving direction at the foot end of the driving leg.

**Figure 6:**
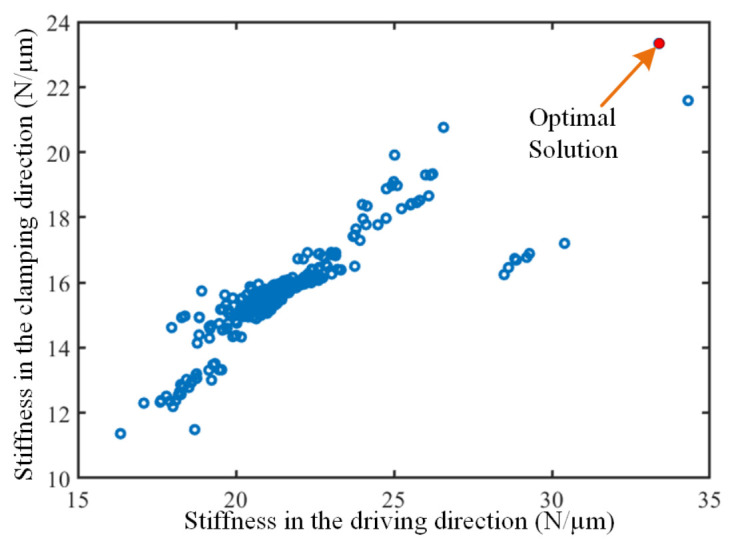
Optimal solution set of the flexible mechanism.

The NCGA algorithm possesses both full search capabilities and local generalization capabilities in multi-objective optimization, and it has a fast convergence speed. Therefore, this work adopts the NCGA algorithm for optimization. After 600 iterative calculations, the solution set is obtained, as shown in Figure 6. According to the principle of the maximum stiffness in the clamping direction and the driving direction, the optimal solution selected from the solution set is marked with red circles. The stiffness in the clamping direction of the driving leg is 23.33 N/µm, and the stiffness in the driving direction is 33.39 N/µm. The lengths of the corresponding nine key design variables are *L*_1_ = 10.7874 mm, *L*_4_ = 3.676 mm, *L*_5_ = 10.86525 mm, *H*_2_ = 9.994 mm, *H*_6_ = 6.4722 mm, *H*_8_ = 1.6047 mm, *H*_9_ = 2.8357 mm, *H*_10_ = 4.34 mm, and *H*_11_ = 5.682 mm, respectively.

Considering the actual processing technology level, the convenience of assembly and the compactness of the structure, the dimensions of the nine key design variables are adjusted or fine-tuned based on the analysis results of the main effect. The final values of each design variable are *L*_1_ = 10.6 mm, *L*_4_ = 3.6 mm, *L*_5_ = 10.9 mm, *H*_2_ = 10 mm, *H*_6_ = 6.5 mm, *H*_8_ = 0.6 mm, *H*_9_ = 1.9 mm, *H*_10_ = 3.3 mm, and *H*_11_ = 4.7 mm. Considering the compactness of the structure, *L*_4_ was reduced to 1.6 mm. As it is a symmetrical structure, the corresponding size of *L*_1_ is 6.6 mm. The simulation results show that the displacement in the clamping direction of the flexible mechanism, as well as the stiffness in the clamping and driving directions, are slightly reduced at this size, but still meet the design requirements. In addition, the research in work [58] indicates that an appropriate combination of friction pairs is conducive to reducing wear and improving the stability of actuator driving. However, considering the actual processing, this work selects the friction pair combination of bearing steel (GCr15) and zirconia (ZrO2). Through the finite element analysis software, the displacement in the clamping direction at the foot end of the driving leg under the design parameter can be obtained as 23.11 µm, the stiffness in the clamping direction as 15.41 N/µm, the displacement in the driving direction as 8.83 µm, and the stiffness in the driving direction as 17.59 N/µm (the previous text is the simulation data of the rhombic mechanism, so the stiffness in the clamping direction and the driving direction of the driving leg is smaller than that of the rhombic mechanism). The following text will verify the effectiveness of the design method through experimental tests of the displacement and the stiffness in the clamping direction of the driving leg and the smooth movement of the actuator.

## 4. Experiments and Discussion

### 4.1. Characterization of Driving Legs

**Figure 7:**
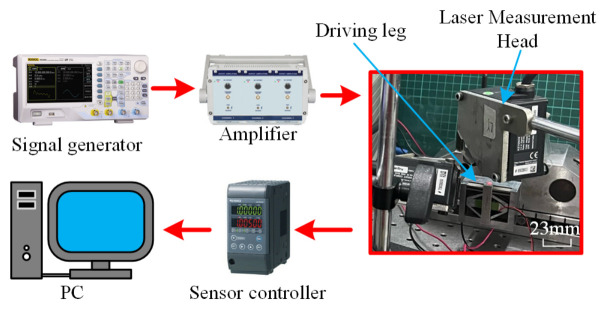
Driving-leg foot end displacement testing device.

To test the foot end displacement of the driving leg, an experimental setup as shown in Figure 7 was established. The excitation signal generated by the signal generator was amplified 30 times by the power amplifier (E00. A3, Coremorrow, Harbin, China) and then sent to the piezoelectric stack or ceramic of the driving leg. The motion data at the foot of the driving leg was received by the laser displacement sensor (LK-H020, Keyence, Osaka City, Japan) and recorded on the PC.

**Figure 8:**
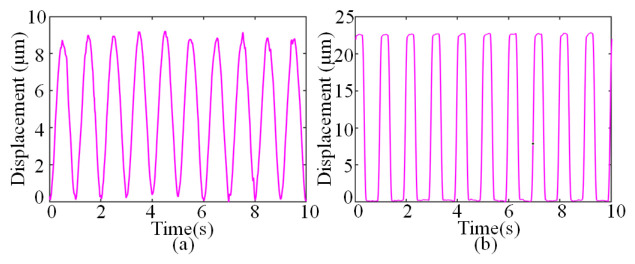
The displacement characteristics of the driving leg: (**a**) Displacement in the driving direction. (**b**) Displacement in the clamping direction.

The displacements in the foot end of driving direction and clamping direction were recorded by applying a 300 V_p-p_ sinusoidal voltage on the piezoelectric ceramic (driving direction) and a 150 V trapezoidal voltage on the piezoelectric stack, respectively. Through the analysis and organization of the data recorded in the experiment, Figure 8 and Table 2 are obtained; the numbers in the table represent the serial numbers of different driving legs, and the numbers in Table 3 also represent the serial numbers of different driving legs. Under the simulation conditions, the displacements at the foot end of the driving leg in the driving direction and the clamping direction were 8.83 µm and 23.11 µm, respectively. The maximum deviation of the displacement in the driving direction of the six driving legs from that under the simulation conditions is 6.5%, and the maximum deviation of the displacement in the driving direction between the six driving legs measured in the experiment is 9.3%. The maximum deviation of the displacement in the clamping direction of the driving legs from that under the simulation conditions is 3.9%. The maximum deviation of the displacement in the clamping direction between the six driving legs measured experimentally is 3.2%. The push-pull force gauge was respectively extruded in the clamping direction and the driving direction of the driving-leg foot end. The displacement changes in the extrusion head were recorded with a laser displacement sensor, and the equivalent stiffness value in the clamping direction at the foot end and the driving direction was obtained as shown in Table 3. The maximum deviation between the measured equivalent stiffness value in the driving direction and the designed equivalent stiffness value is 7.8%, and the maximum deviation between the measured equivalent stiffness value in the clamping direction and the designed equivalent stiffness value is 8.6%. Both deviations are within the allowable range. The deviation between the experimental results and the simulation results is mainly caused by the following factors: (1) the material properties actually processed by the prototype deviate from those in the simulation; (2) there are deviations between the piezoelectric coefficients of piezoelectric stacks and piezoelectric ceramics from the simulated values; and (3) there are differences in the simulation values of the driving legs assembled in the experiment. The deviation between the experimental test results and the simulation results is within an acceptable range. Therefore, comparing the experimental and simulation values in the driving direction and the clamping direction can verify the effectiveness of the design method. In the subsequent research, the actual material properties, the piezoelectric coefficients of stack and the piezoelectric coefficients of the ceramic will be used as simulation parameters to simulate the actuator, to obtain smaller simulation calculations and provide more accurate guidance for driver design.

### 4.2. Displacement Resolution of Actuator

**Figure 9:**
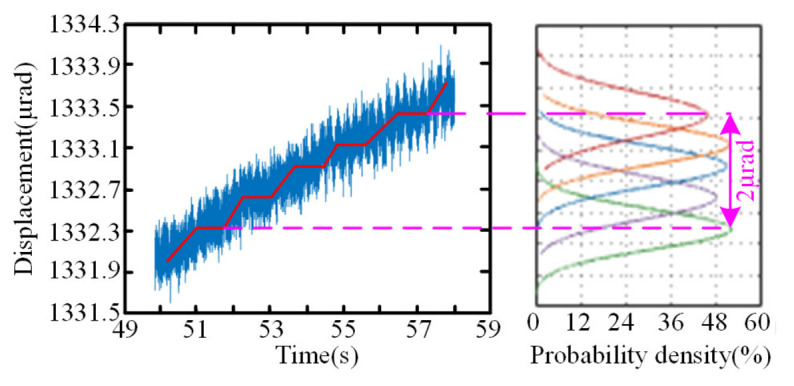
The resolution of the actuator. In the left part of the figure, the blue line is the original data, and the purple line is the fitted data. in the right part of the figure, different colors represent the data of differet steps.

When the voltage amplitude of 15 V_p-p_ is applied horizontally to the actuator, and the voltage is divided into five steps, the displacement curve as shown in Figure 9 can be obtained. The five steps move a total of 2 µrad, so the resolution of the actuator is 0.4 µrad.

### 4.3. Smoothness Characterization of Actuators

**Figure 10:**
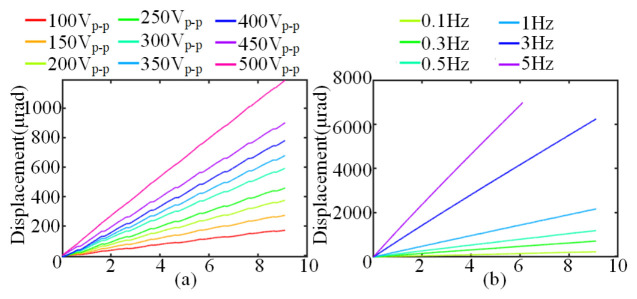
Displacement characteristics of the actuator: (**a**) Displacement characteristics under different voltage amplitudes. (**b**) Displacement characteristics at different voltage frequencies.

Keeping the pre-pressure between the foot end of the driving leg and the rotor constant, and maintaining the voltage frequency in the driving direction at 0.5 Hz, the displacement curve obtained by changing the voltage amplitude in driving direction is shown in Figure 10a. Keeping the pre-pressure between the foot end of the driving leg and the rotor constant, maintaining the voltage amplitude in driving direction at 500 V_p-p_, and changing the voltage frequency in driving direction, the displacement curve obtained is shown in Figure 10b.

As can be seen from Figure 10a,b, the displacement under different excitation conditions changes linearly with time. Through analysis, it is found that the linear fitting coefficient of all displacement under the excitation conditions in Figure 10a,b reaches above 0.999. Therefore, it can be determined that the actuator has achieved smooth motion. Through calculation, the speed of the actuator under the excitation conditions of voltage amplitude of 100 V_p-p_ and actuation frequency of 0.5 Hz is 18.83 µrad/s, and the speed of the actuator under the excitation conditions of voltage amplitude of 500 V_p-p_ and actuation frequency of 5 Hz is 1144.38 µrad/s.

### 4.4. Load Characteristic Testing

**Figure 11:**
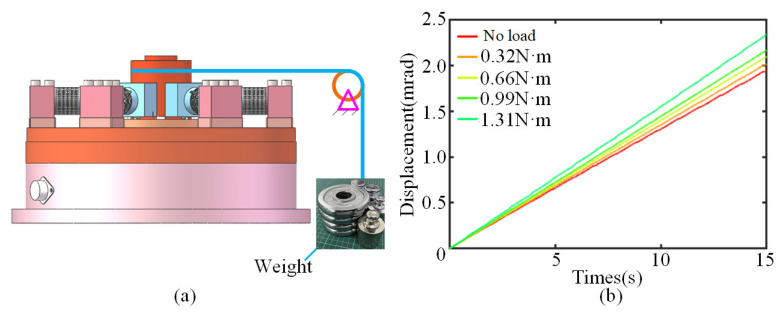
Load characteristic test of the actuator: (**a**) Schematic diagram of the load characteristic test device. (**b**) Displacement under different load torques.

The voltage amplitude of 500 V_p-p_ and the excitation frequency of 0.5 Hz were applied in the horizontal direction of the actuator. The heavy objects with masses of 0 kg, 1 kg, 2.06 kg, 3.12 kg and 4.12 kg were suspended on the output shaft, respectively, as shown in Figure 11a. The output displacement of the actuator was obtained as shown in Figure 11b. The difference in output displacement under heavy objects of different masses is mainly due to the change in contact stiffness between the driving leg and the rotor caused by the heavy object, resulting in the difference in the displacement of the driving leg in the driving direction. The linear fitting coefficient of all displacement curves under different suspended objects is 0.9999, demonstrating excellent smoothness. The output shaft diameter of the actuator is 65 mm. When the 4.12 kg load is suspended, the corresponding output torque is 1.31 N·m. Currently, the actuator still maintains good smoothness. Therefore, the output torque of this actuator is better than 1.31 N·m.

### 4.5. Application Demonstrations

**Figure 12:**
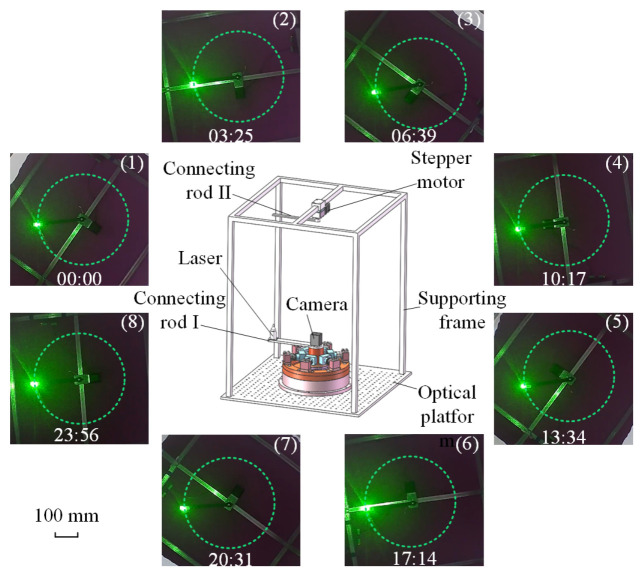
The tracking experimental setup and the experimental results.

To verify the application potential of the proposed multi-legged walking-type smooth piezoelectric actuator in the field of deep space exploration, a demonstration experiment of tracking application was carried out. The experimental setup is shown in Figure 12. This setup consists of a multi-legged walking-type smooth piezoelectric actuator, connecting rod I, laser, camera, supporting frame, connecting rod II, stepper motor, etc. The multi-legged smooth-motion walking-type piezoelectric actuator and the supporting frame are all installed on the optical platform. Connecting rod I is installed on the output shaft of the actuator, the laser is installed on connecting rod I, the stepper motor is installed on the supporting frame, and connecting rod II is installed on the output shaft of the stepper motor. During the tracking experiment, the actuator rotates the laser, and the stepper motor rotates at the same speed as the actuator to ensure that the light signal of the laser always falls on the connecting rod II.

**Figure 13:**
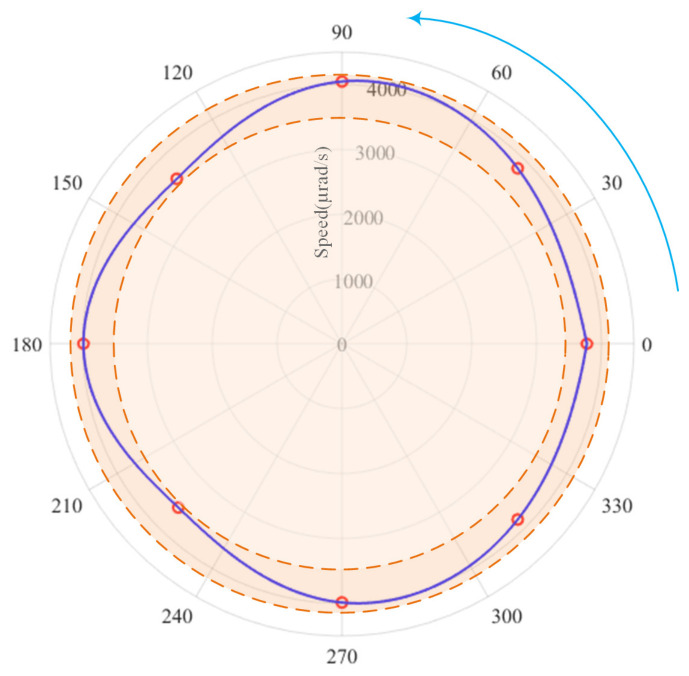
Experimental data analysis of the tracking demonstration.

The actuator was made to rotate one entire cycle, and the process was recorded by a camera. Data points were selected every 45° for analysis. The experimental results are shown in Figure 12, and the analysis results of each experimental data point are presented in Figure 13. The experimental results indicate that at each experimental position, the laser on the actuator was incident on connecting rod II, achieving excellent tracking and aiming performance. The data analysis results show that when the actuator is in operation, the running interval between adjacent test points is 205 s, the corresponding average speed is 3829.27 urad/s, and the speed fluctuation of the actuator during the entire-cycle operation was between −5.4%~7.2%, suggesting that the actuator has the ability to perform smooth entire-cycle motion; it can be determined that the actuator can achieve smooth motion within the speed range of 18.83 µrad/s to 3829.27 µrad/s. Furthermore, this demonstration experiment was conducted after the basic-characteristics and torque-characteristics experiments were completed, with an interval of more than one year. However, the actuator still maintained excellent output characteristics. The experimental results show that choosing the appropriate friction pair is beneficial for improving the friction interface of the actuator and achieving good long-term durability. Therefore, this multi-legged walking-type smooth-motion actuator has application potential in the field of deep-space exploration.

### 4.6. Performance Comparison

The comparison of this rotary actuator with other rotary piezoelectric actuators is shown in Table 4. The rotating piezoelectric actuator proposed in this paper not only achieves smooth movement, but also has the output torque reaching the N·m level, even surpassing the output torque of many ultrasonic rotating piezoelectric actuators. Moreover, its resolution is higher than that of stick-slip actuators and inchworm actuators. More importantly, its maximum smooth movement speed is the lowest among all the drives, and its minimum smooth movement speed can reach 18.83 µrad/s. These characteristics are of great significance in the field of deep space exploration.

## 5. Conclusions

This work presents a smooth-motion rotary piezoelectric actuator over a large travel range with high torque based on the principle of the bipedal walking mechanism. By imitating the walking gait of humans, it alternately fuses the actuation trajectories of two sets of driving legs with high stiffness and large displacement, adjusts the balance between the driving torque and the resistance torque, and ensures that the resultant force torque acting on the rotor is zero, thereby achieving smooth motion over a large travel range with high torque. The NCGA multi-objective optimization algorithm was adopted to optimize the stiffness and displacement of the driving leg, the high torque output was achieved by optimizing the stiffness of the driving leg, and the smooth motion was achieved by optimizing the displacement in the clamping direction to disengage the driving leg from the rotor over a large travel range. The displacement in the clamping direction of the driving leg obtained through simulation was 23.11 μm. The maximum deviation between the experimental value and the simulation value of the displacement in the clamping direction of the driving leg was 3.9%, and the maximum deviation between the experimental value and the simulation of the stiffness value in the clamping direction was 8.6%. The displacement of the driving leg in the driving direction obtained by simulation was 8.83 μm. The maximum deviation between the experimental value and the simulation value of the displacement in the driving direction of the driving leg was 6.5%, and the maximum deviation between the stiffness experiment and the simulation in the driving direction was 7.8%. The deviations were all within the allowable range, which preliminarily verifies the effectiveness of the design method. The actuator was driven by a multi-legged alternating driving mode, the linear fitting coefficients of the output displacement of the actuators all reached above 0.999, the resolution of the actuator was 0.4 µrad, and the output torque of the actuator was better than 1.31 N·m. In the optical tracking demonstration application experiment, this experiment was conducted under quasi-static and stationary conditions. The speed fluctuation of the whole tracking and aiming was −5.4% to 7.2%, which is more able to reflect the physical meaning of motion smoothness compared to relying only on the linear fitting coefficient (R^2^). At the same time, compared with other studies, it further highlights the advantage of this work in achieving smooth motion and large torque within the large travel range. These outstanding performances will greatly expand the application scope of piezoelectric actuators, especially in scenarios where there are high requirements for the torque and motion smoothness of the actuators. The multi-legged stepping piezoelectric actuator performs significantly better than the single-legged stepping piezoelectric actuator. However, it is difficult to keep the characteristics of different driving legs consistent. In the future, we will focus on standardizing the assembly process of the actuator to enhance the research and development efficiency of the multi-legged stepping piezoelectric drive. At the same time, we will explore the environmental adaptability of this actuator in actual engineering applications.

## Figures and Tables

**Table 1:** Structural parameters of the flexure hinge actuator.

Symbols	Parameters	Values
*L*_1_	Internal length of the root	5 mm
*L*_2_	External length of the upper arm	13.5 mm
*L*_3_	Internal length of the upper arm	11.8 mm
*L*_4_	Length of the stacking installation seat	3.5 mm
*L*_5_	Internal length of the lower arm	11.5 mm
*L*_6_	External length of the lower arm	14.3 mm
*L*_7_	External length of the root	3 mm
*H*_1_	Internal height of the root	1 mm
*H*_2_	External height of the upper arm	9.3 mm
*H*_3_	Internal height of the upper arm	8 mm
*H*_4_	Height of the stacking installation base	6.5 mm
*H*_5_	Internal height of the lower arm	5.5 mm
*H*_6_	External height of the lower arm	6.5 mm
*H*_7_	Height of the foot tip	9.2 mm
*H*_8_	Height of the upper arm of the first crossbeam	1.5 mm
*H*_9_	Height of the lower arm of the first crossbeam	2.8 mm
*H*_10_	Height of the upper arm of the second crossbeam	4.3 mm
*H*_11_	Height of the lower arm of the second crossbeam	5.6 mm
*R*_1_	Outer fillet of the upper arm	0.5 mm
*R*_2_	Internal fillet of the upper arm	0.3 mm
*R*_3_	Outer fillet of the lower arm	0.5 mm
*R*_4_	Internal fillet of the lower arm	0.3 mm
*W*_1_	Thickness of the first crossbeam	6.6 mm
*W*_2_	Thickness of the second crossbeam	6.6 mm

**Table 2:** Displacements in driving versus clamping directions of the six driving legs.

	Serial Number	1	2	3	4	5	6
Evaluation Object							
Horizontal (µm)		8.6	8.9	9.4	9.4	8.7	8.8
Deviation		2.6%	0.8%	6.5%	6.5%	1.5%	0.3%
Vertical (µm)		22.4	22.2	22.9	22.2	22.8	22.6
Deviation		3.1%	3.9%	0.9%	3.9%	1.3%	2.2%

**Table 3:** Equivalent stiffness value in driving versus clamping directions of the six driving legs.

	Serial Number	1	2	3	4	5	6
Evaluation Object							
Horizontal (N/µm)		16.32	16.21	16.38	16.58	16.73	16.67
Deviation		−7.2%	−7.8%	−6.9%	−5.7%	−4.9%	−5.2%
Vertical (N/µm)		14.08	14.35	14.56	14.62	14.29	14.18
Deviation		−8.6%	−6.8%	−5.5%	−5.1%	−7. 3%	−7.9%

**Table 4:** Comparison between rotary piezoelectric actuators.

References	Type	Size (mm^3^)	Smooth Motion	Maximum Speed (mrad)	Output Torque (N·m)	Resolution (µrad)
[27]	Ultrasonic	Ø40 × 110	/	12,664.67	10.1	/
[59]	Ultrasonic	70 × 46 × 34	/	6489.33	0.86	6.7
[60]	Ultrasonic	/	/	26,690	0.3	/
[47]	Stick-slip	84 × 84 × 33	Yes	1042.99	0.045	1.77
[51]	Stick-slip	150 × 85 × 30	Yes	1452	/	25
[61]	Inchworm	Ø80 × 25	No	6.51	0.0931	4.95
[62]	Inchworm	55 × 60 × 13.5	No	26.74	0.245	4.79
Proposed	Multi-legged	Ø320 × 179	Yes	3.83	>1.31	0.4

## Data Availability

The datasets generated and analyzed during the current study are available from the corresponding author upon reasonable request.

## Supplementary Materials

The following are available online at https://www.mdpi.com/article/10.3390/biomimetics11070495/s1, Video S1: structural configuration, working principles, and demonstration experiment of the actuator.
